# Development of muscle atrophy and loss of function in a Gulf-War illness model: underlying mechanisms

**DOI:** 10.1038/s41598-020-71486-w

**Published:** 2020-09-03

**Authors:** Israel Ramirez-Sanchez, Viridiana Navarrete-Yañez, Alejandra Garate-Carrillo, Maria Loredo, Esmeralda Lira-Romero, Javier Estrada-Mena, Anaamika Campeau, David Gonzalez, Marvic Carrillo-Terrazas, Aldo Moreno-Ulloa, Guillermo Ceballos, Francisco Villarreal

**Affiliations:** 1 School of Medicine, UCSD, 9500 Gilman Dr. BSB4028, La Jolla, CA 92093-0613 USA; 2 Seccion de Estudios de Posgrado e Investigacion, Escuela Superior de Medicina, IPN, Mexico City, Mexico; 3 Escuela de Medicina, Universidad Panamericana, Mexico City, Mexico; 4 Departamento de Innovación Biomédica, CICESE, Mexico, Mexico; 5 VA San Diego Health Care, San Diego, CA USA

**Keywords:** Energy metabolism, Fatigue

## Abstract

Gulf War illness (GWI) afflicts military personnel who served during the Persian Gulf War and is notable for cognitive deficits, depression, muscle pain, weakness, intolerance to exercise, and fatigue. Suspect causal agents include the chemicals pyridostigmine (PB), permetrim (PM) and N,N-diethyl-m-toluamide (DEET) used as protectants against insects and nerve gases. No pre-clinical studies have explored the effects on skeletal muscle (SkM). Young male rats were provided PB, PM and DEET at equivalent human doses and physical restraint (to induce stress) for 3 weeks followed a 3-week recovery. GWI gastrocnemius weight was ~ 35% lower versus controls, which correlated with decreases in myofiber area, limb strength, and treadmill time/distance. In GWI rats, SkM fiber type relative abundance changed towards slow type I. Muscle wasting pathway proteins were upregulated while those that promote growth decreased as did mitochondrial endpoints and muscle ATP levels. Proteomic analysis of SkM also documented unique alterations in mitochondrial and metabolic pathways. Thus, exposure to GWI chemicals/stress adversely impacts key metabolic pathways leading to muscle atrophy and loss of function. These changes may account for GWI Veterans symptoms.

## Introduction

Gulf War Illness (GWI) afflicts ~ 30% of the US military personnel who served in the 1990–1991 first Persian Gulf War. GWI comprises a gathering of symptoms that prominently affect the nervous and skeletal muscle (SkM) systems leading to cognitive deficits, muscle pain, weakness, exercise intolerance and fatigue^1,2^. GWI affected Veterans continue to experience symptoms and altered function after 25+ years. However, the clinical presentation of GWI is unique to the 1990–1991 conflict, with no similar illness being reported in any other military campaign, indicating that the etiology cannot solely be attributed to combat-related stress^2^.

While the precise etiology is unknown, several hypotheses have been proposed most prominently, co-exposure to specific chemical agents and stress^2^. Military personnel stationed in the battlefield are believed to have consumed the acetylcholinesterase inhibitor (AChEi) pyridostigmine bromide (PB) pills as a daily prophylactic treatment to protect against nerve gas ^1,2^. In addition, to reduce the risk of infections transmitted by vectors, personnel were also exposed to insecticides and insect repellants most commonly permethrin (PM) and N,N-dietyl-m-toluamide (DEET). PM is a widely used insecticide and intoxication leads to the opening of voltage-gated sodium channels^3^. DEET is also widely used and its target is unknown, but human poisoning can occur. AChEi, organophosphate toxicity and lethality have been related to the development of oxidative stress (OS) and mitochondrial dysfunction (MD)^2–4^.

Rodent models have been developed to examine the effects that GWI associated chemicals and stress have on physiological systems. A significant amount of pre-clinical work has focused on the nervous system as GWI Veterans commonly suffer from neurological impairments. Exposure of rodents to equivalent human doses of PB, PM and DEET, and stress triggers multiple neurological perturbations which have been linked to changes in brain structure and function^5–8^. However, as noted above, fatigue is a prominent symptom of the disease and the SkM system has not been examined for the possible adverse impact of GWI associated chemicals as such compounds can lead to MD and ATP depletion. In fact, a recent clinical study detected a prolongation of SkM phosphocreatine recovery time in GWI Veterans after a bout of lower limb exercise leading to the hypothesis of MD as a key disease mechanism^4^. In a separate report, the analysis of white blood cells in GWI Veterans also detected mutations in mitochondrial DNA further supporting the MD hypothesis^9^.

The objective of this study was to use an established rat model of GWI and evaluate the effects that chemicals and stress exposure trigger on SkM structure and function. Furthermore, we examined the possible roles that MD and activation of atrophy related pathways play in the development of this pathology.

## Results

**Figure 1:**
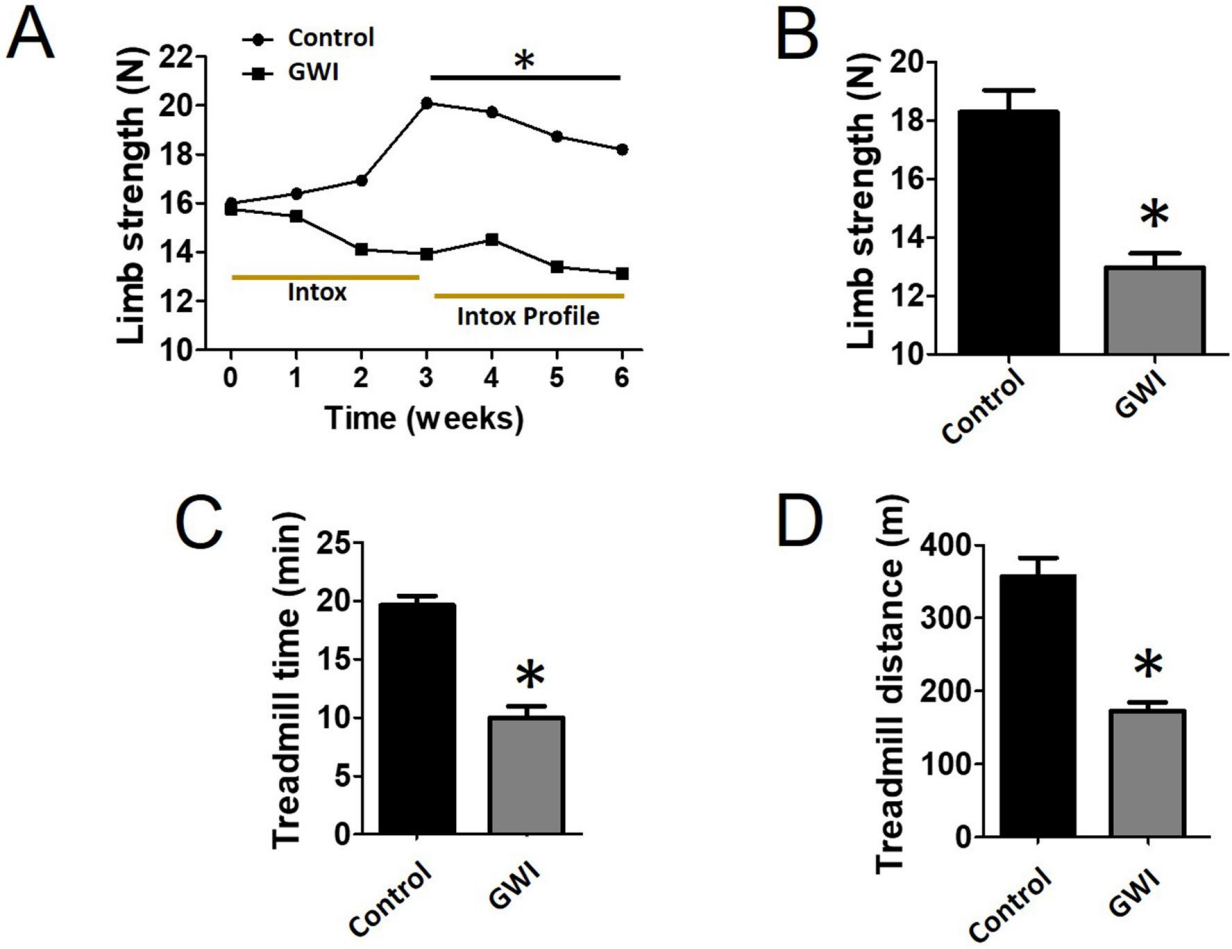
Analysis of muscle function. Front limb strength recorded weekly (**A**) and at the final (6 week) recording (**B**) in control (n = 18) and GWI (n = 17) rats. Panels (**C**,**D**) report on the treadmill test results (time and total distance respectively). *p < 0.05 ANOVA and by t-test.

**Figure 2:**
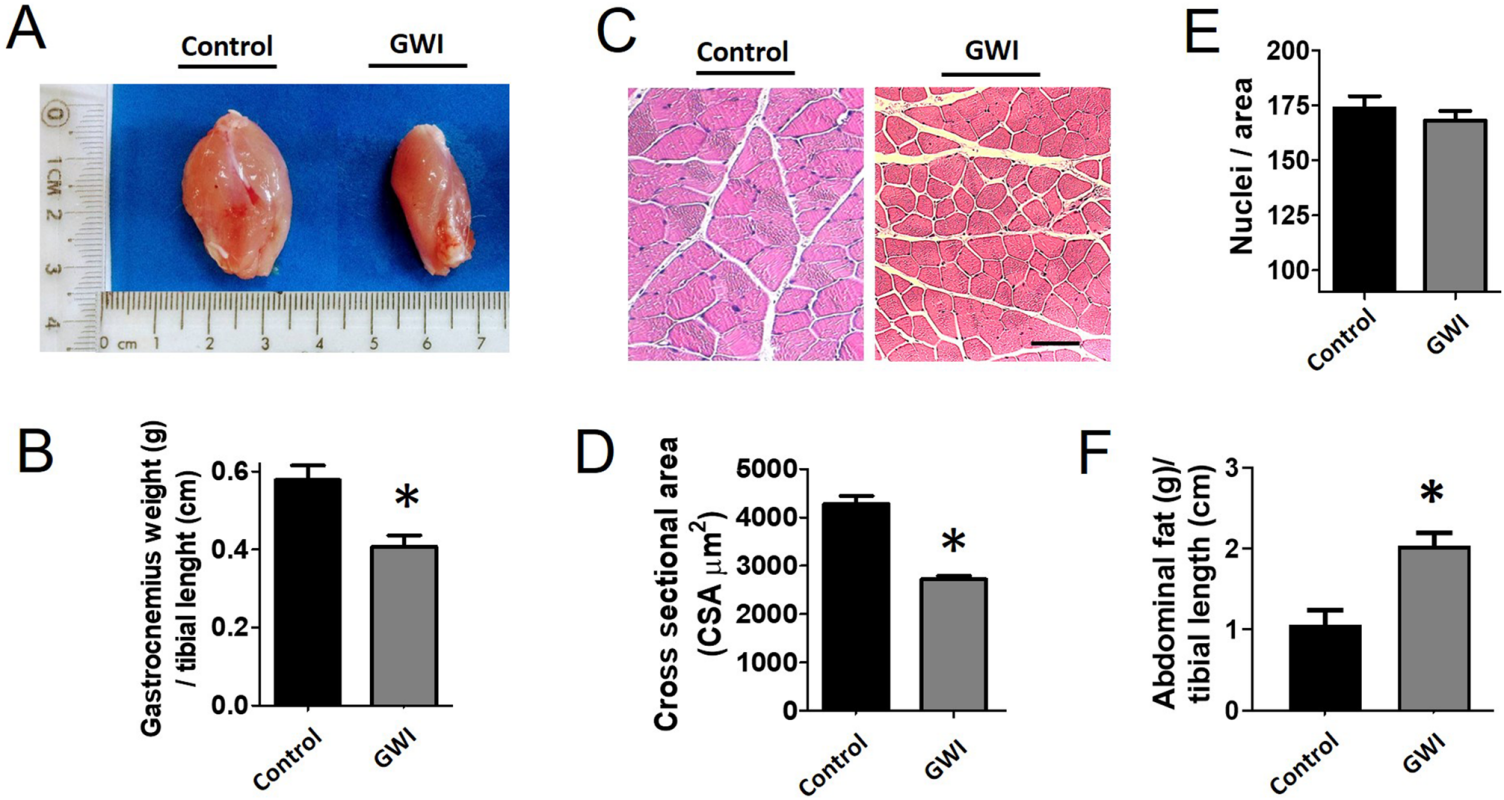
Effects of exposure on gastrocnemius muscle mass. Panels (**A**,**B**) illustrate and report on weight recorded for gastrocnemius muscles in control (n = 18) and GWI rats (n = 17). Panel (**C**) are images recorded from muscle stained with hematoxilin and eosin (scale bar = 50 µm). Panel (**D**) reports on myofiber area measured by image analysis and panel (**E**) on the corresponding number of cell nuclei per area. Panel (**F**) reports on weight of abdominal fat in control (n = 18) and GWI rats (n = 17). *p < 0.05 by t-test.

**Figure 3:**
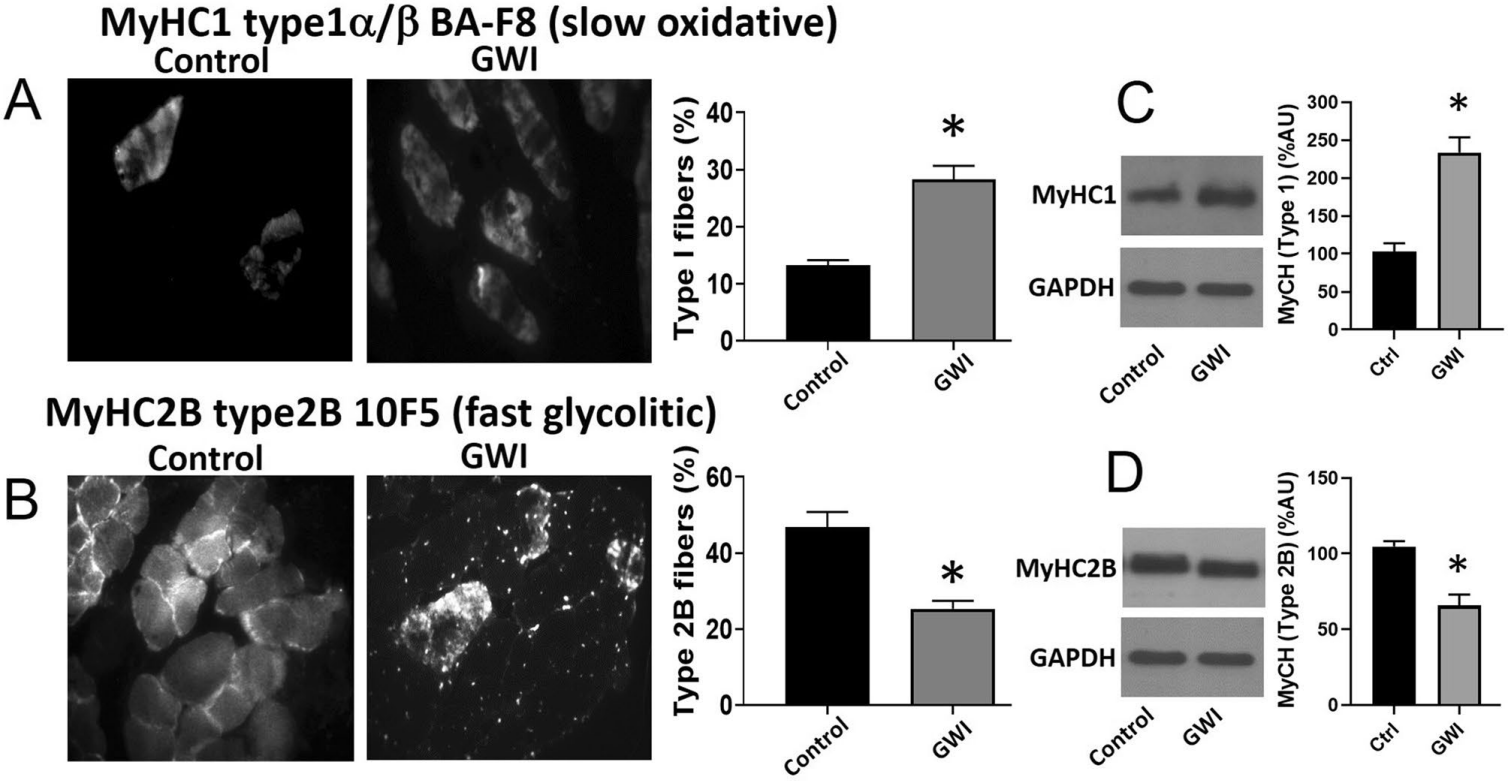
Effects of exposure on gastrocnemius myofiber isotype. Frozen tissue sections were stained with antibodies directed against slow (**A**) or fast (**B**) myosin heavy chains. Bar graph report on the percentage of positively stained fibers normalized by imaged control versus GWI animals (n = 7/group). Western blot detection of myosin heavy chains slow (**C**) or fast (**D**) are shown in the upper bands. Bar graphs report on the quantification of relative protein amount normalized by percentage of GAPDH (lower bands) in control and GWI animals (n = 6/group). *p < 0.05 by t-test.

**Figure 4:**
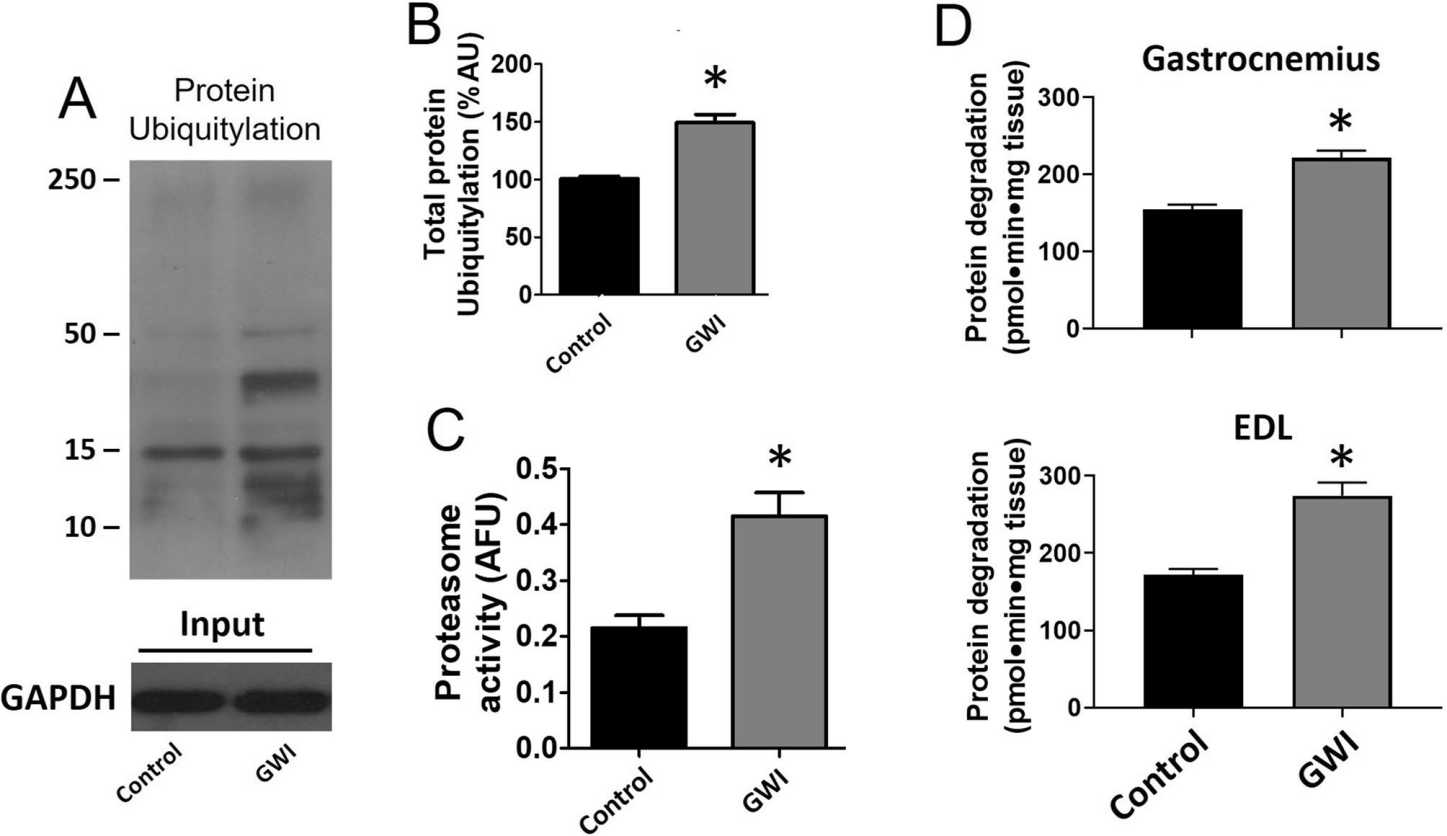
Effects of exposure on gastrocnemius muscle protein ubiquitylation and degradation. Panels (**A**) is an immunoblot image illustrating enhanced levels of muscle total protein ubiquitylation whereas panel (**B**), reports on the relative quantification in control and GWI animals (n = 6/group, AU = Arbitrary Units). Panels (**C**) reports on the quantification of proteasome activity (arbitrary fluorescent units = AFU) and panel (**D**) reports on protein degradation by tyrosine release in gastrocnemius and EDL muscles. *p < 0.05 by t-test.

Food intake for both groups of animals was comparable for the duration of the study (28.6 ± 2.7 in controls vs. 29.5 ± 1.9 g/day in GWI). The final body weight recorded was also similar (414 ± 19 in controls vs. 402 ± 20 g in GWI). Figure 1A plots average once/week recorded front limb strength. While control animals demonstrated an increase in strength at the 3-week time point, GWI animals demonstrated a steady progressive decline that was significantly different from weeks 3–6. The final strength recorded at week 6 is plotted in panel B and shows ~ 28% drop in GWI animals. Treadmill time and distance are reported in panels C and D respectively, demonstrating significant decreases of ~ 50% and ~ 66% respectively versus controls. Figure 2 reports on changes in SkM mass (A, B), myofiber cross-sectional area (C, D), myofiber nuclei numbers (E) and abdominal fat weight (F). A significant loss of ~ 34% in muscle mass was recorded in gastrocnemius, which correlated with a comparable decline in myofiber cross-sectional area, while nuclei numbers were unchanged. Extensor digitorum longus (EDL) muscle mass was also recorded, evidencing a decreased of ~ 30%, which is comparable to that observed in gastrocnemius. Additionally, an ~ 45% gain in abdominal fat weight was recorded. Figure 3 illustrates comparable changes detected in myofiber isotypes relative abundance as per slow type 1 and fast type 2B myosin heavy chain immunostaining (A, B) and Western blot (C, D). In GWI animals, there was a significant percentage change in myofiber type from fast (type 2B) to slow (type 1). Figure 4 illustrates gastrocnemius total protein ubiquitylation (A, B), proteasome activity (C) and protein degradation (tyrosine release) (D) gastrocnemius (upper panel) EDL (lower panel) results. Panel A is a representative image of a Western blot used to document/quantify enhanced protein ubiquitylation in GWI samples with quantification in panel B. Proteasome activity and protein degradation results (gastrocnemius and EDL) also indicate significant increases in GWI samples.

**Figure 5:**
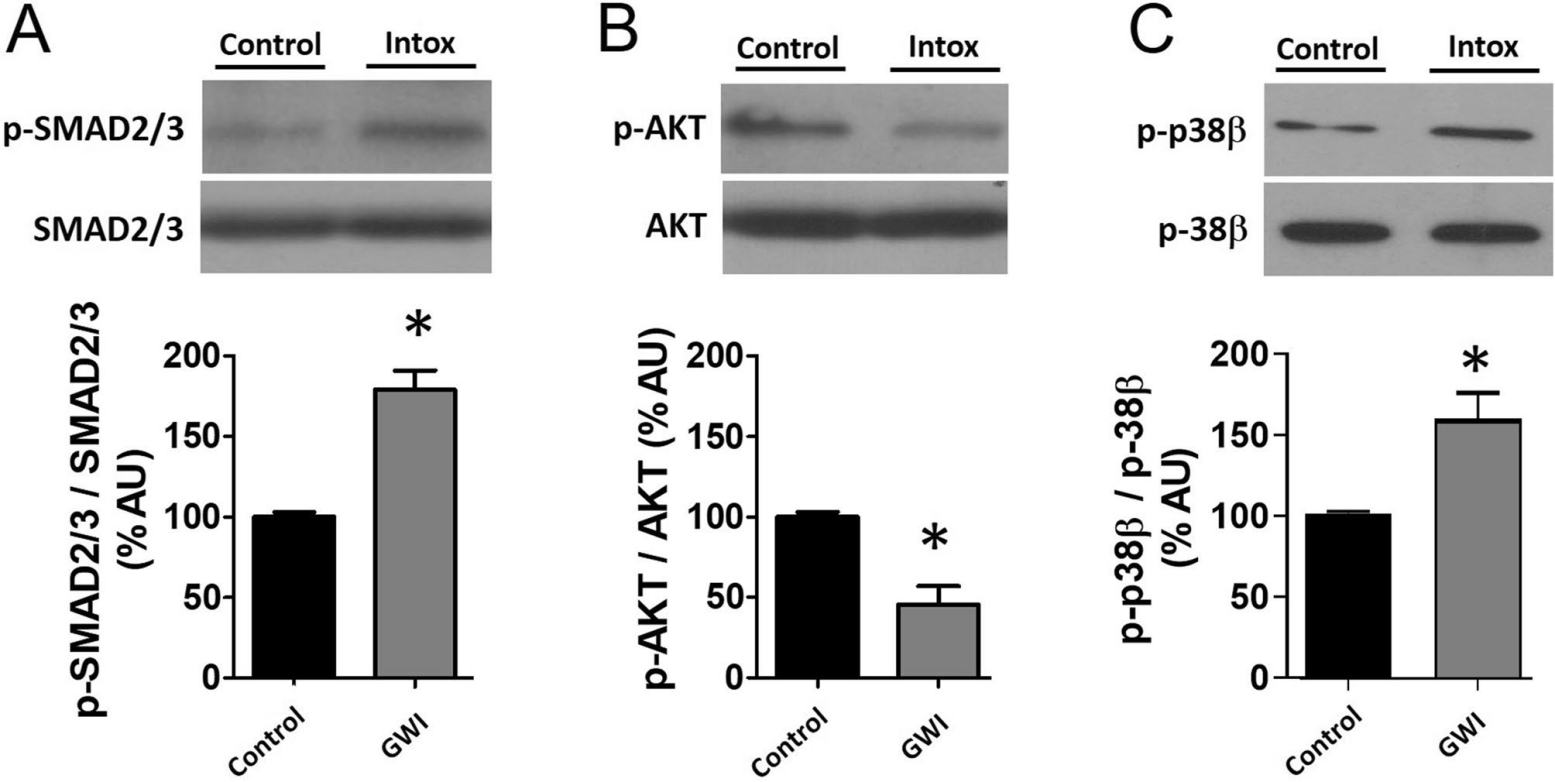
Effects of exposure on upstream muscle mass regulator pathways. Panel (**A**) reports on the effects of exposure on SMAD2/3 phosphorylation levels as detected by immunoblots in control and GWI animals (n = 6/group) while panel (**B**) reports on the phosphorylation of AKT and C, p38β. *p < 0.05 by t-test. AU = Arbitrary Units.

**Figure 6:**
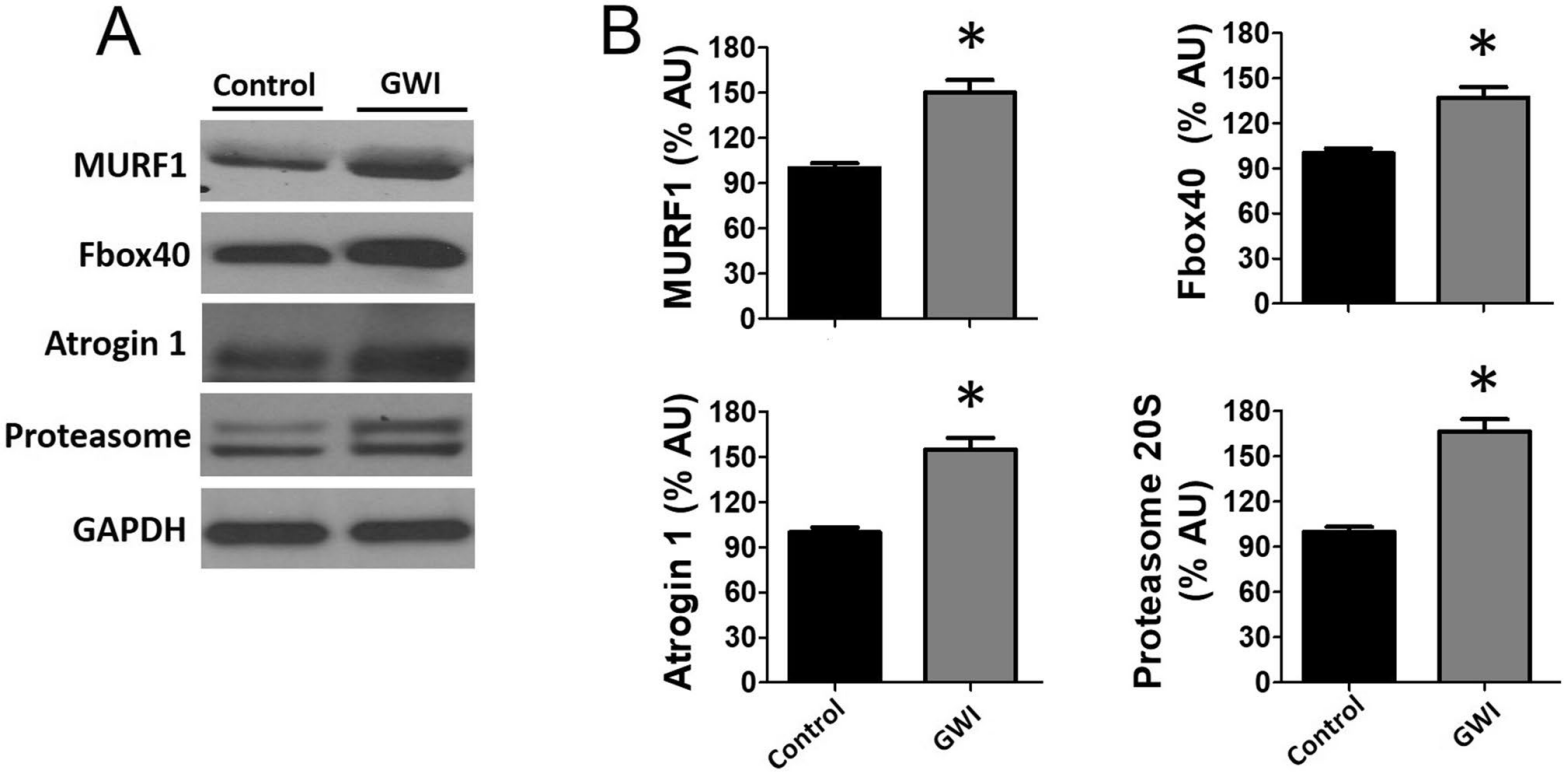
Effects of exposure on modulators of muscle atrophy. Panel (**A**) illustrates a representative image and panel (**B**) reports on relative changes in protein levels of gastrocnemius Murf1, Fbox40, atrogin1 and proteasome 20S in control and GWI animals (n = 6/group). *p < 0.05 by t-test. AU = Arbitrary Units.

**Figure 7:**
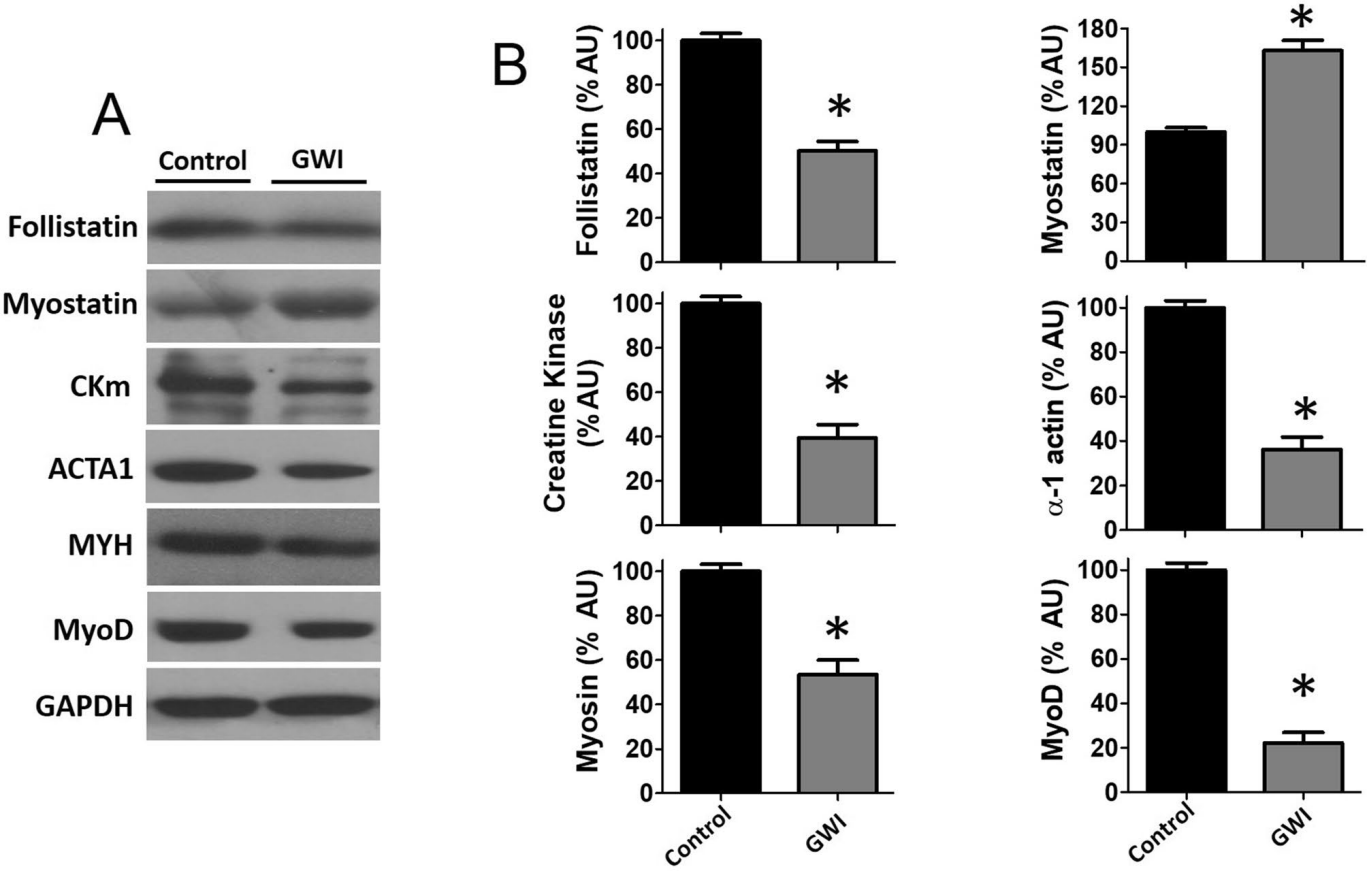
Effects of exposure on modulators of muscle mass and constitutive proteins. Panel (**A**) illustrates a representative image and panel (**B**) reports on relative changes in protein levels of gastrocnemius follistatin, myostatin, muscle creatine kinase, α1-actin, myosin heavy chain 2a and MyoD in control and GWI animals (n = 6/group). *p < 0.05 by t-test. AU = Arbitrary Units.

Figure 5 documents the involvement of muscle atrophy signaling pathways. Western blots of gasctrocnemius GWI muscle demonstrate the significant activation of p-Small Mothers Against Decapentaplegic (SMAD) 2/3 (A) while suppressing that of phospho protein Kinase B (p-AKT) (B) and activation (phosphorylation) of p-38β MAP kinase (C). Figure 6A reports on the assessment of regulatory systems involved in muscle atrophy, including Muscle RING finger 1 (MURF1), Muscle Atrophy F box (MAFbx)40, atrogin 1, and proteasome 20S. Significant increases in relative protein levels were observed for all four factors in GWI samples (Panel B). Figure 7A reports changes in relative protein levels for regulators of muscle development [Myogenic Differentiation (MyoD)], muscle mass (follistatin, myostatin), and constitutive proteins (myosin, α1-actin and muscle creatine kinase). GWI demonstrated a significant upregulation of myostatin while reducing the levels for all the other proteins, thus suggesting that muscle atrophy includes the coordinated involvement of multiple control systems (B).

**Figure 8:**
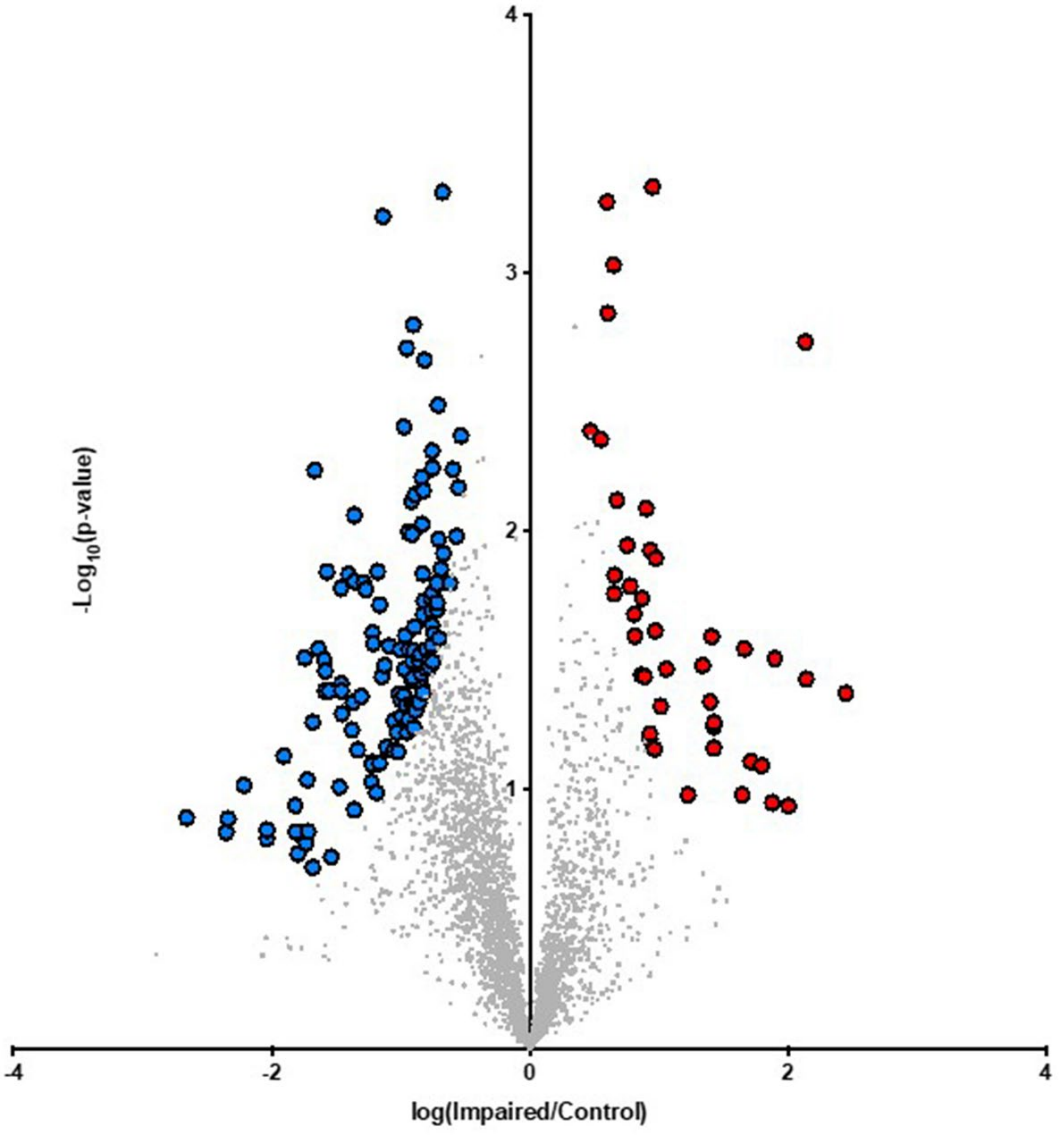
Volcano plot on the effects of exposure on gastrocnemius proteasome profile. Upregulated proteins of control (n = 4) and GWI animals (impaired, n = 5) are plotted to the right of the vertical axis, whereas those downregulated are on the left.

**Figure 9:**
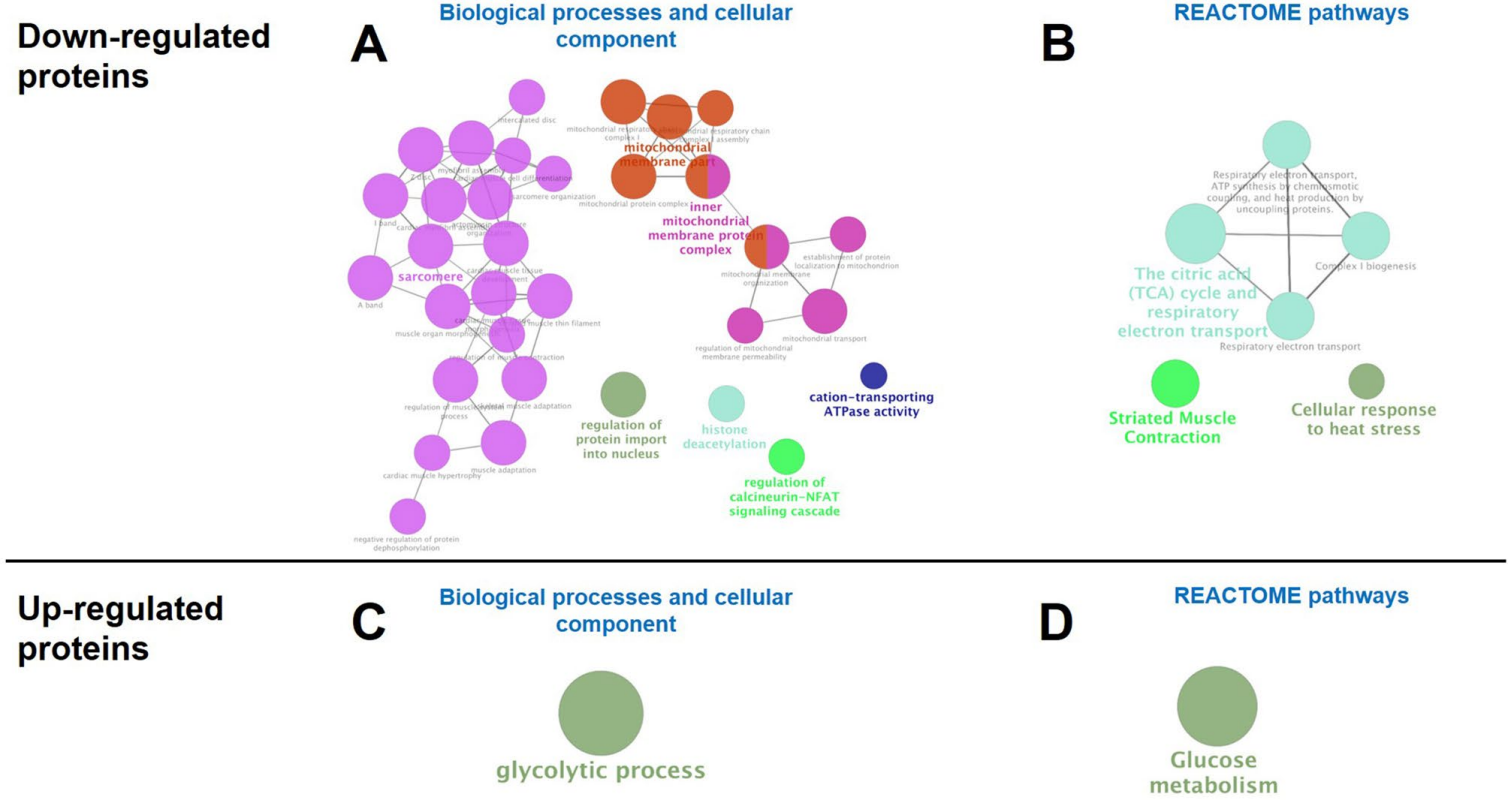
Gene ontology (GO) enrichment analysis of differentially abundant proteins in GWI animals. Biological processes and cellular components associated with downregulated (**A**,**B**) and upregulated (**C**,**D**) proteins. The name of the most significant term in each group is shown. GO enrichment analyses were performed using ClueGo app for Cytoscape (version 3.7.0). An enrichment/depletion (two-sided hypergeometric test) method with Benjamini–Hochberg correction was performed. A minimum and maximal GO level of 3 and 8 were used, respectively. Kappa Score was set to 0.4.

**Figure 10:**
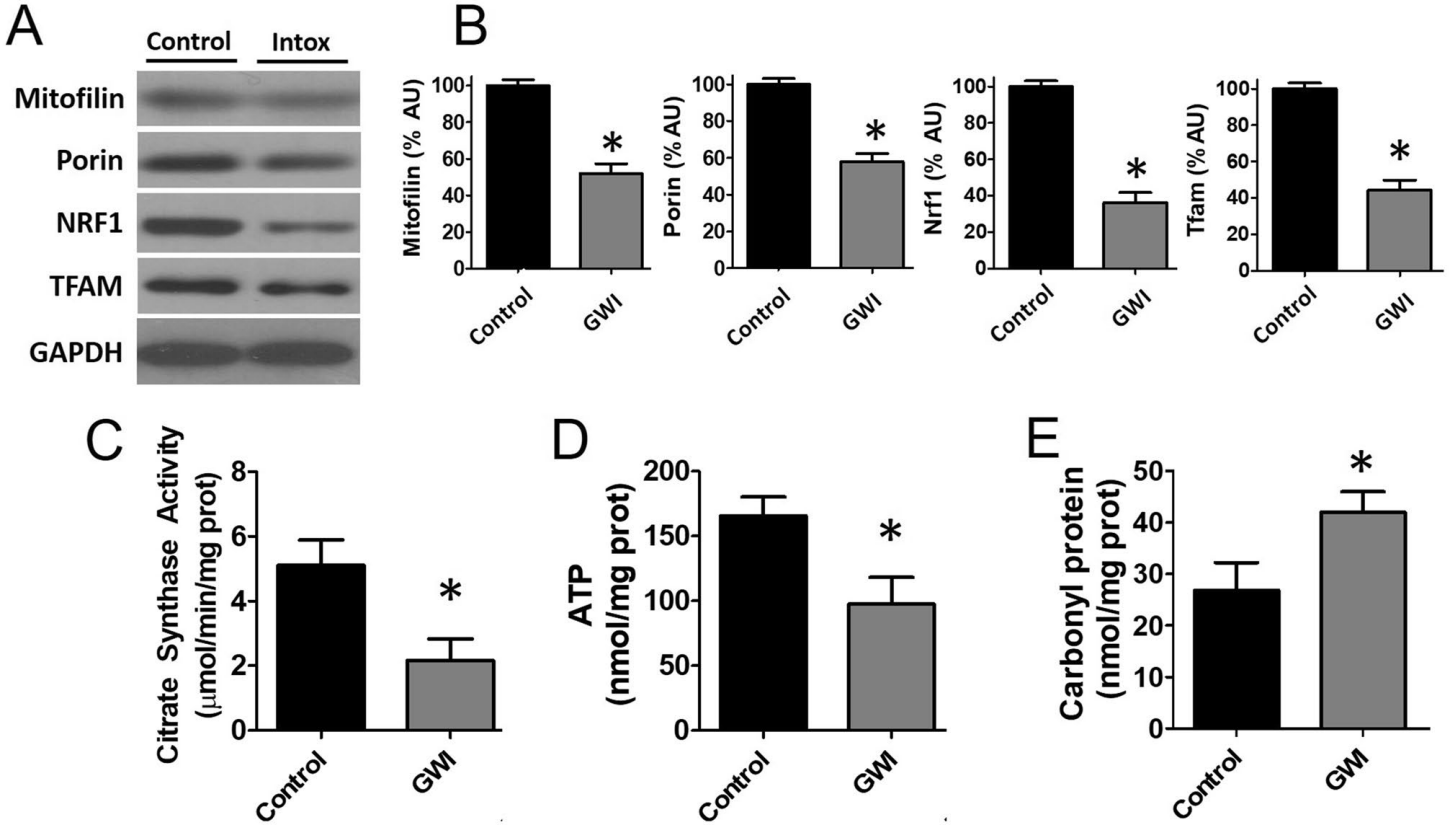
Effects of exposure on muscle mitochondrial enzymatic function and oxidative stress levels. Panels (**A**) illustrate representative Western blots images and panel (**B**) reports on relative changes in protein levels of gastrocnemius mitofilin, porin, Nrf1 and Tfam in control and GWI animals (n = 6/group). Panels (**C**–**E**) report on citrate synthase activity, ATP and plasma protein carbonyl levels respectively. *p < 0.05 by t-test. AU = Arbitrary Units.

Proteomic analysis of control and GWI muscle samples revealed and quantified 3026 proteins in total. Binary comparison of GWI versus control muscle demonstrated 42 and 126 proteins that were up- and down-regulated at a significance threshold of pi score > 1.1082. Results from the binary comparison and significance threshold imposition are illustrated using a volcano plot (Fig. 8). Those that were significantly lower are denoted by blue symbols (left half of plot) while upregulated proteins are represented in red (right half of the plot). More proteins were downregulated during challenge with GWI-inducing agents versus upregulated. Gene ontology enrichment analyses were performed on down-regulated and up-regulated proteins. Enrichment analysis by biological processes and cellular component annotations, as well as the REACTOME-associated pathways is illustrated in Fig. 9, wherein the label of the most significant term per group is shown. For down-regulated proteins, enrichment analysis indicates the apparent involvement of proteins linked to cellular components of the sarcomere and mitochondrial structures, as well as to the biological processes of regulation of protein import into the nucleus, histone deacetylation, cation-transporting ATPase activity, and regulation of calcineurin-NFAT signaling cascade (A). Associated REACTOME pathways were found for the mitochondria, skeletal muscle, and cellular response to heat stress (B), while upregulated proteins were linked to glucose metabolism (C and D), which suggest that the process of muscle atrophy is closely linked to mitochondrial abnormalities. As mitochondria appeared to be notably affected in GWI animals, samples of muscle were analyzed for changes in relative protein levels for mitofilin (a cristae protein), porin (outer membrane protein) and the regulators of mitochondrial biogenesis, Nuclear regulatory factor (Nrf1) and Mitochondrial transcription factor A (Tfam) (Fig. 10A,B). In all cases, significant decreases in protein levels occurred, ranging from ~ 40 to 60% of controls. To assess for possible changes in mitochondrial function we used the indirect indicators citrate synthase activity and ATP levels (C,D). In line with changes detected in mitochondrial protein levels, citrate synthase activity decreased by ~ 60%, while ATP levels had a significant reduction of ~ 40% in GWI muscle samples. These data correlated with an upregulation of total protein carbonyl levels of ~ 30% in gastrocnemius (E) suggesting a possible state of MD.

## Discussion

Unique findings from this study provide evidence for a major involvement of SkM in the development of a GWI like disease in rodents exposed to suspect chemicals and stress. Exposure to GWI chemicals led to the activation of muscle atrophy, which was documented at recognized signaling pathways, downstream modulators, constitutive proteins, and fiber size and type. The analysis of mitochondria related endpoints document detrimental changes in those that regulate biogenesis, structure, and enzymatic function while linking these shifts with high SkM oxidative stress levels. These alterations were accompanied with a selective loss of the predominant fast type 2B fibers and loss of muscle function. Altogether, changes in SkM suggest that major symptoms recognized in GWI Veterans likely involve this system.

GWI is a syndrome that in its early stages was difficult to recognize and validate as a unique pathology as no similar disease had emerged in previous conflicts. Stress as a causal element remained for many years a prominent suspect as many of the symptoms can potentially be triggered by it. As epidemiological evidence for a unique disease became stronger and suspected environmental elements, including co-exposure to multiple chemicals, emerged, the implementation of animal models followed. Co-exposure to the chemicals PB, DEET, and PM became most suspect, given their wider use in the Gulf War theater^2^. Initial studies were implemented in hens using “high” doses of PB, PM, and DEET and were published in 1996^10^. Then, studies in rats^11^ using lesser doses of chemical were performed, wherein altered locomotor activity was reported. These studies principally established that exposure to the three chemicals appeared critical to the development of adverse effects. As the most prominent complaints of GW veterans included chronic fatigue, muscle pain, brain-related abnormalities, ataxia, inability to concentrate, forgetfulness, and behavioral symptoms alterations in the nervous and SkM systems became suspect^1^. Indeed, previous publications recognized the potential for PB, DEET and PM (when given individually) to trigger neurotoxic effects^10^. Thus, most pre-clinical GWI studies initially focused on the impact that co-exposure to these agents had on neurological systems.

The first comprehensive assessment of the effects that combined chemical exposure and mild stress had on neurological endpoints was published in 2002 by Abdel-Rahman et al.^3^ In this study, rats were exposed daily for 28 days to a combination of stress and chemicals (at doses relevant to troop exposure which are the same used in this study). Animals exhibited blood–brain barrier disruption and neuronal cell death in the cingulate cortex, dentate gyrus, thalamus, and hypothalamus. Liver histology demonstrated sinusoidal dilatation, microvacuoles, inflammatory infiltrate, and periportal fibrosis. Only until recently did animal models explore the effects that stress and chemicals have on behavior measures. Parihar^12^, demonstrated in rats an association between depressive- and anxiety-like behavior, spatial learning and memory dysfunction with decreased hippocampal neurogenesis, loss of neurons and inflammation. Thus, an apparent clear link was established between detrimental changes in neurological structures and function many of which are associated with similar symptoms in GW Veterans. The results of these studies were further corroborated by recent studies using rodents^13,14^. Additional pre-clinical studies reported on alterations in other organs such as the colon^15^. Interestingly, in spite of fatigue being a prominent symptom in GW Veterans, the SkM system had not been a subject of focused examination.

Recent findings for the possible involvement of MD have emerged from studies in GW Veterans. MD emerged as a possible mechanistic link as poisoning with organophosphate agents such as PM had been associated with organelle dysfunction and oxidative stress triggering symptoms including fatigue, brain, gastrointestinal, sleep, and autonomic dysfunction, which are commonly seen in GWI^7^. Phosphocreatine is a storage energy source present in SkM that is depleted during exercise, and the speed of recovery is dependent on the rate of mitochondrial ATP synthesis. Post-exercise phosphocreatine recovery rate has been validated as a measure of MD in vivo^16^. A case–control study compared the phosphocreatine recovery rate in Veterans by Ref.^31^phosphorus magnetic resonance spectroscopy with GWI matched controls. A significant prolongation of the recovery time was detected in GWI Veterans after a bout of lower limb exercise thus, supporting the hypothesis of MD as a putative pathophysiological mechanism^4^. In further support of this hypothesis, are reports of impaired exercise induced VO_2_ max in GWI Veterans^17^. It was also recently noted that in peripheral blood, mononuclear cells of GWI Veterans (after 25+ years) retain evidence of greater mitochondrial DNA damage versus controls, which associated with decreased complex I and IV enzymatic activities^9^. Interestingly, a pilot clinical trial tested the effects of mitochondrial coenzyme Q10 versus placebo in GWI Veterans and control subjects^18^. Improvements were noted in global indicators of physical function and symptoms, suggesting that targeting mitochondrial “health” can provide a benefit to GWI patients.

In spite of the preponderance of evidence suggesting SkM involvement no pre-clinical studies had examined the impact that the combined use of chemicals and stress had on organ structure and function. Exposure of rats to the same doses of chemicals and stress as published by others, led to a progressive decline in muscle strength that became more evident in the 2nd week of exposure and continued at a milder pace until 3 weeks after exposure to reach ~ 13 N in strength versus ~ 18 N in controls (~ 28% in strength). A significant impairment in treadmill time and total distance ensued. A rather unexpected finding was the degree of muscle atrophy detected in gastrocnemius as well as in EDL muscle, which was evident, by simple visual inspection. The degree of atrophy observed was corroborated by CSA measures, loss of SkM α-actin, myosin heavy chain, and creatine kinase. It is also possible, that the loss of muscle mass is the result of an inhibition of protein synthesis. Accompanying atrophy, was the switching of the predominant type of myosin present in gastrocnemius from fast (2B) to the slow type. Altogether, a process that encompasses a major loss of muscle mass and fiber type switching was evident that is similar to that reported to occur with hindlimb immobilization. These changes occurred while preserving total body weight and maintaining normal food intake but, with a notable increase in abdominal fat.

Myostatin is recognized as the most potent negative regulator of muscle mass growth and activates the atrophy process by binding to cell membrane receptors that link to the activation of the SMAD 2/3 and p38b pathways^19,20^. Follistatin is an effective antagonist of myostatin and exerts such an effect by binding to myostatin and blocking receptor docking^21^. Both factors are produced in muscle and other tissues and circulate in blood. Thus, the analysis of changes in their levels can provide a pathway for muscle atrophy development. Indeed, the analysis of changes in relative protein levels indicated an ~ 70% upregulation in myostatin with a 50% drop in follistatin. These changes aligned with evidence for the activation of SMAD 2/3 and p38b as per its enhanced phosphorylation and concomitant suppression of the AKT pathway which is known to preserve/promote muscle mass^19–21^.

The process of muscle atrophy is complex. However, key regulators have been recognized and validated, which include those involved in modulating the activity of the ubiquitin–proteasome system that is required to remove sarcomeric proteins^19^. Atrophy associated events include, (1) the conjugation of ubiquitin to muscle proteins, (2) increased proteasomal activity, (3) increased protein breakdown and, (4) stimulation of ubiquitin-conjugating enzymes (ligases). Three prominent muscle-specific ubiquitin ligases include atrogin-1, FBXO40, and MuRF1^19^. Immunoblot analysis demonstrated the upregulation of protein levels for both ligases in gastrocnemius samples in the range of 30–60%. Atrogin-1 is known to promote the degradation of MyoD and we detected a decrease of 80% in gastrocnemius protein levels^19^. These changes correlated with increases in proteasome 20S protein levels and atrogin 1 suggesting increased activity of systems associated with protein labeling/processing for degradation^19^. Indeed, the analysis of gastrocnemius protein demonstrated increased total protein ubiquitylation. Using a protein degradation assay (as per tyrosine release) we also demonstrate increased degradation of gastrocnemius and EDL protein in the range of ~ 25–45%%, which may be indicative of a selective loss of fast fibers. Thus, evidence supports the active engagement of atrophy mediator pathways, which remained involved 3 weeks after exposure^19^.

To better understand the comprehensive nature of the changes induced by exposure, a proteomic analysis of gastrocnemius was performed. An overall preferential downregulation of total proteins detected (3026) was evident in the volcano plot. Forty proteins reached the pre-established significance threshold for downregulation, whereas only 18 were upregulated. Upregulated proteins are associated to altered cell metabolism. A systems analysis of downregulated proteins identifies changes in muscle sarcomere structure, protein import into nucleus, calcium signaling, epigenetic regulatory mechanisms of SkM function, and mitochondrial structure and transport. Mitochondrial function and density are closely linked to muscle health. The development of muscle atrophy correlates with the remodeling of the mitochondrial network and the activation of autophagy. When mitochondrial structure and/or function are altered/blocked using pharmacological (e.g. with chloramphenicol, tetracyclines or rifampicin) or molecular strategies (e.g. ethidium bromide) muscle differentiation is impeded in vitro and in vivo^22^. Conversely, SkM regeneration is associated with increased citrate synthase activity in differentiating cells and the upregulation of mitochondrial biogenesis factors such as Tfam and Nrf1^23^. The analysis of gastrocnemius GWI samples detected significant decreases ~ 40–65% in Tfam and Nrf1 as well in the cristae and outer membrane proteins mitofilin and porin which correlated with detrimental changes in indirect indicators of mitochondrial function as per reduced citrate synthase activity, ATP levels, and increases detected in plasma carbonyl levels. Altogether, results derived from this study provide a clear link between MD and impaired muscle performance, as seen in GW Veterans. The likely role played by inflammation and cytokines such as tumor necrosis factor-α in muscle atrophy remains to be determined as these factors appear to be involved as per results from pre-clinical and clinical studies.

The extent reported here for muscle atrophy is somewhat without precedent for exposure to chemical agents. For decades it has been known that glucocorticoids induce muscle atrophy in the range of ~ 20% at maximal doses^19,24^. The pathways linked to such effects parallel those described above^19^. However, treatment of animals with dexamethasone at atrophy inducing doses also triggers significant weight loss, which we did not detect in GWI rats^24^. Thus, more studies need to address the potential of GWI suspect agents to either disrupt mitochondria and/or activate atrophy inducing pathways. A recent publication reported on the effects that 4-weeks exposure to chemicals/stress had on rat hippocampus 6 months after exposure^25^. Results implicate the involvement of inflammation, oxidative stress, and MD in alterations in memory and mood. Thus, the participation of mitochondria appears critical to disease development.

Results from this study for the first time document the likely involvement of the SkM in GWI disease development. These findings, in conjunction with those reported in neurological and other systems, suggest a broader involvement in disease development, which warrants further studies in particular long-term as GW Veterans continue to be adversely impacted by the disease. The capacity of the tested agents to adversely impact muscle structure/function warrants further consideration of their toxicological potential.

## Methods

### Study design and animal model

In brief all experimental protocols were approved by a UCSD’s Research Committee licensing committee and all methods were carried out in accordance with relevant guidelines and regulations.

Our GWI animal model is based on a model developed by Hattiangady et al. ^13^, with minor modifications. In brief, 3 month old, male Wistar rats underwent 3 weeks of exposure to PB (oral gavage 1.3 mg/kg/day) PM and DEET (skin applications 0.13 and 40 mg/kg/day respectively, in 70% ethanol) as well as physical restraint (stress) by placing the animals in a plexiglass holder for 5 min/day (n = 17) ^12^. Control animals were given only vehicles and no restraint (n = 18). Animals were then allowed to recover for 3 weeks. Food intake and body weight were recorded every other day during the exposure and recovery period. After their final functional assessment, animals were euthanized and tissues collected, including abdominal fat, which were weighed.

### Muscle strength

Front limb muscle strength was measured once/week using a grip-strength meter device. Animals are placed on a metal grid where their front paws hold onto a front T-bar. Upon the sudden application of a tail-pull, the animals in a reflex manner pull the bar and tension is recorded digitally. Measurements were performed 3 times and averaged.

### Treadmill testing

Tests were performed as published by us. Rats were initially familiarized with the treadmill device at a slow speed (~ 5 m/min) at 10° incline for ~ 10 min for 2 days previous the final test. Once familiarized, the exhaustion test consisted of a warm-up at 4 m/min for 2 min followed by an increase of 2 m/min every min thereafter. A shock grid and air jets at the back of the treadmill were used to discourage animals from stopping. Exhaustion was defined as when rats were no longer able to maintain their normal running position and/or were unwilling to run as indicated by contact with the shock grid, which was readily deactivated. Running time was recorded and total distance calculated.

### Histomorphometry

Quadriceps muscles were weighed and trimmed before their immediate formaldehyde fixing or freezing in glycol/resin media for subsequent sectioning. Once sectioned, muscle samples were stained with Hematoxilin and Eosin and microscope imaged to digitally quantify nuclei and myofiber cross-sectional area (a total of 450 fibers/group were measured). Samples were also immunostained to ascertain for the presence of myosin heavy chain fast type 2B (fast glycolytic) and slow type 1 fiber isotype (slow oxidative). EDL muscles were also weighed and preserved frozen. Abdominal fat tissue was collected and weighed.

### Western blotting

To assess the effects of exposure on signaling pathways associated with the regulation of muscle atrophy and relevant downstream endpoints, relative protein levels for Akt, phospho-Akt, SMAD2/3, phospho-SMAD2/3, MURF1, Fbox40 (Cell Signaling), atrogin 1 (Abcam) and proteasome S20 (Santa Cruz Biotechnology)were determined in samples of gastrocnemius muscle. For fiber isotype determination and relative protein level determinations, myosin heavy chain slow (type 1) and fast (type 2B) (BA-F8 and 10-F5 respectively, Developmental Studies Hybridoma Bank) were evaluated. For regulators of muscle differentiation and growth as well as constitutive proteins, changes in protein levels for MyoD, follistatin, myostatin (Abcam), muscle creatine kinase (ThermoFisher Scientific), myosin heavy chain, and p38β (Santa Cruz Biotechnology), phospho p38β and α1-actin (ThermoFisher Scientific) were determined. For regulators of mitochondrial biogenesis and structural changes, Nrf1, Tfam, porin, and mitofilin (Abcam) relative protein levels were assessed. Muscle homogenates were prepared and a total of 30 µg of protein were loaded onto a 4–15% gel, electrotransferred, incubated for 1 h in blocking solution (5% non-fat dry milk in tween-Tris buffer saline) and followed by either 3 h incubation at room temperature or overnight at 4 °C with primary antibodies. Primary antibodies were typically diluted 1:1000 or 2000 in tween buffer plus 0.5% bovine serum albumin or 2% milk-based buffer. Membranes were washed (3× for 5 min) with tween buffer and incubated 1 h at room temperature in the presence of species-specific horseradish peroxidase-conjugated secondary antibodies diluted 1:5000 in blocking solution. Membranes were again washed 3 times with tween buffer and immunoblots developed using chemiluminescence. Band intensities were digitally quantified and normalized using D-Glyceraldehyde-3-Phosphate Dehydrogenase (GAPDH) as a loading control.

### Biochemical assays

#### Protein degradation

The protein degradation rate was measured as net tyrosine release from isolated muscle samples. Gastrocnemius and EDL muscle samples (10 mg) were preincubated for 30 min in Krebs Ringer buffer [NaCl 1.2 mmol/L; KCl 4.8 mmol/L; NaHCO_3_ 25 mmol/L; CaCl_2_ 2.5 mmol/L; KH_2_PO_4_ 1.2 mmol/L and MgSO_4_ 1.2 mmol/L; pH 7.4], supplemented with glucose [5.5 mmol/L], bovine serum albumin [1.0 g/L], insulin [5 U/mL], and cyclohexamide [5 mmol/L], saturated with 95% O_2_/5% CO_2_ gas mixture. Muscles were transferred into a fresh medium of the same composition and incubated for 2 h. At the end of the incubation, medium samples were used to measure tyrosine released by spectrophotometry.

#### Protein ubiquitylation

Skeletal muscle lysates were prepared by homogenizing gastrocnemius (15 mg) in a buffer. Samples were centrifuged at 12,000 g for 10 min and supernatants collected. A total of 30 µg of protein were loaded onto a 4–15% gel and transferred to polyvinylidene fluoride membranes. An antibody (Abcam) was used for the detection of ubiquitinated proteins by immunoblotting.

#### Proteasome activity assay

Proteasome activity was measured by using a Kit (Abcam) that detects chymotrypsin-like activity using a 7-amino-4-methylcoumarin tagged peptide substrate that generates a fluorescent product in the presence of proteolytic activity. Lysates of gastrocnemius muscle (15 mg) were used. Homogenates were centrifuged at 12,000 g for 10 min and supernatants collected. Proteasome activity was measured with fluorescent substrates of the tagged peptide. The assay was conducted in the absence and presence of the specific proteasome inhibitor MG-132 to determine proteasome-specific activity. Released tagged peptide was measured using a fluorometer at an excitation wavelength of 350 nm and emission of 440 nm.

#### Citrate synthase

Muscle samples were homogenized in a buffer and 20 μg of protein was used. Enzymatic activity was determined by a colorimetric assay kit based on the reaction between 5′,5′-Dithiobis 2-nitrobenzoic acid (DTNB) and CoA-SH to form TNB, which exhibits maximum absorbance at 412 nm. The enzyme activity was determined in duplicates and read by a spectrophotometer at 412 nm, wherein the absorbance is proportional to enzyme activity.

#### SkM protein carbonylation

Protein carbonylation was measured in gastrocnemius (15 mg) using an assay for the detection of carbonyl groups that relies on 2,4-dinitrophenylhydrazine as a substrate. The reaction leads to the formation of a stable 2,4-dinitrophenyl hydrazine product, which is quantified spectrophotometrically at 375 nm.

#### SkM ATP levels

ATP levels were measured in 5 mg of liquid nitrogen frozen gastrocnemius muscle that was pulverized and then homogenized in the kit buffer. A commercial luciferase-based bioluminescence kit was used to quantify ATP levels (Cayman Chemical, Inc. #700410). Tissue homogenates were diluted in sample buffer and used to measure ATP levels within 10 min after luciferase reaction as per instructions using a luminometer.

### Proteomic analysis

#### Sample preparation for proteomic analysis

Gastrocnemius samples were placed in a lysis buffer comprised of 75 mM NaCl, 3% SDS, 1 mM NaF, 1 mM β-glycerophosphate, 1 mM Na_3_VO_4_, 10 mM Na pyrophosphate, 1 mM phenylmethylsulfonyl fluoride, 1X complete EDTA-free protease inhibitor cocktail, and 50 mM HEPES, pH 8.5 and 500 μl of 8 M urea with 50 mM HEPES, pH = 8.5. Ceramic beads were added to the fill line of the sample tubes and samples were subjected to bead-beating with a Mini-Beadbeater-24. Bead-beating was performed at 4 °C for three cycles of 1 min shaking intercalated with 1 min of rest. Homogenized samples were separated from the ceramic beads and the resulting homogenate was subjected to probe sonication with a Q500 QSonica sonicator equipped with 1.6 mm microtip. Sonication was performed at 20% amplitude for 3 cycles of 15 s intercalated with 10 s of rest. Disulfide bonds were reduced in 5 mM of dithiothreitol at 56 °C for 30 min. Sample tubes were cooled on ice for 5 min. Reduced disulfide bonds were alkylated in 15 mM of iodoacetamide in a darkened environment at room temperature for 20 min. The alkylation reaction was quenched in 5 mM dithiothreitol in a darkened environment at room temperature for 15 min.

Protein was precipitated using a chloroform–methanol precipitation protocol described previously. Briefly, 6 ml of methanol, 1.5 ml of chloroform, and 4.5 ml of HPLC-grade water were added to sample tubes. The sample solution was vigorously agitated through vortexing and tube inversion. Samples were then subjected to centrifugation at 4000 rpm for 2 min at room temperature. The supernatant was removed, and 6 ml of methanol was added to the sample tubes. The samples were vortexed and subjected to centrifugation at 4000 rpm for 2 min at room temperature. The supernatant was removed from samples and the precipitated protein was kept on ice for the remaining washes. Samples were washed with 600 μl of ice-cold acetone, subjected to vigorous vortexing, and spun for 2 min at 4000 rpm at 4 °C. The supernatant was discarded, and the acetone wash was repeated once more. The resultant precipitated protein was dried at 56 °C for 30 min. The pellet was rehydrated through addition of 900 μl of 1 M urea with 50 mM HEPES, pH = 8.5. The pellet was subjected to 5 min of vortex agitation on a tube shaker and 5 min of water bath sonication.

Digestion of proteins was performed in two steps^26^. In the first, 6 μg of LysC was added to the sample tubes and tubes were allowed to incubate overnight at room temperature on a shaker. In the second digestion step, 5.7 μg of sequencing-grade modified trypsin was added to sample tubes, and then samples were incubated at 37 °C for 6 h. The digestion reaction was quenched through the addition of 40 μl of 10% trifluoroacetic acid. Samples were desalted using previously described methods on C18 columns^27^. The eluted desalted peptide was dried under vacuum. Dried specimens were re-suspended in 1 ml of a solution of 50% acetonitrile and 5% formic acid. Total peptide yield was quantified using the Pierce Colorimetric Quantitative Peptide Assay. Fifty μg of each sample was separated for further analysis and dried under vacuum^28,29^.

Dried 50 μg aliquots were resuspended in 50 μl of a solution of dry acetonitrile with 200 mM of HEPES, pH = 8.5. Samples were vortexed 5 min to ensure complete resuspension of dried peptides. TMT labeling of samples was performed by adding 8 μl of re-suspended TMT label (20 μg/μl) to samples. The labeling reaction was allowed to proceed for 1 h at room temperature. Reaction quenching was performed by adding 9 μl of a 5% solution of hydroxylamine to the sample tubes and allowing samples to incubate at room temperature for 15 min. Samples were acidified through the addition of 50 μl of 1% TFA. Samples were mixed at this stage and desalted on a C18 desalting column using the same previously described protocol. The desalted sample was dried under vacuum.

#### Reverse-phase liquid chromatography

The desalted, labeled, multiplexed sample set was re-suspended in 120 μl of a solution of 5% acetonitrile and 5% formic acid. The sample tube was subjected to vigorous vortexing and water bath sonication for 5 min each. The multiplexed samples were then fractionated on an Ultimate 3000 HPLC with 4.6 mm × 250 mm C18 column. Samples were separated on a gradient progressing from 5 to 90% acetonitrile in 10 mM ammonium bicarbonate for 1 h. Ninety-six fractions were collected. Fractions were concatenated using a previously described method and dried under vacuum^30^.

#### LC–MS/MS/MS

Mass spectrometry-based analysis was performed on an Orbitrap Fusion mass spectrometer with in-line Easy-nLC. Alternating concatenated fractions were used for mass spectrometry-based proteomic analysis. Fractions were suspended in 8 µl of 5% acetonitrile and 5% formic acid, vortexed for 5 min, and sonicated in a water bath for 5 min. Fractions were run on 3-h gradients ranging from 3% acetonitrile and 0.125% formic acid to 100% acetonitrile and 0.125% formic acid. The in-line nLC was connected to the mass spectrometer via in house-packed 30 cm × 100 µm inner diameter, 360 µm outer diameter column comprised of 0.5 cm C4 resin (diameter = 5 µm), 0.5 cm C18 resin (diameter = 3 µm), and 29 cm C18 resin (diameter = 1.8 µm). Ionization of eluted peptides was performed by applying 2000 V of electricity through the T-junction joining sample, waste, and column capillaries. MS1 spectrum acquisition was performed in data-dependent mode with Orbitrap survey scan range of 500–1,200 m/z and resolution of 60,000. Automatic gain control of 2 × 10^5^ was used with maximum ion inject time of 100 ms. MS2 data was collected using the decision tree option. Ions with 2 charges were analyzed between 600 and 1200 m/z, and ions with 3 or 4 charges were analyzed at 500–1200 m/z. The lower limit for the ion intensity threshold was set at 5 × 10^4^. Selected ions were isolated in the quadrupole at 0.5 Th and fragmented via Collision Induced Dissociation. Fragment ion detection and data centroiding occurred in the linear ion trap. The rapid scan rate AGC target was set to 1 × 10^4^.

TMT-based quantitation using MS3 fragmentation was performed. Synchronous precursor selection was used at this stage. Up to 10 MS2 precursors at a time were fragmented using High Energy Collisional Dissociation fragmentation. Reporter ions were detected in the Orbitrap at a resolution of 60,000 and with a lower threshold limit set to 110 m/z. AGC at this stage was set to 1 × 10^5^ with a maximum ion inject time of 100 ms. Data were centroided at this stage and precursor ions more than 40 m/z below and 15 m/z above the MS2 m/z were removed.

#### Proteomics data processing and analysis

Raw spectral data were searched against the reference proteome for Rattus norvegicus (downloaded from https://www.uniprot.org/proteomes/UP000002494 on 5/11/2017) using the Proteome Discoverer 2.0 Software. The Sequest algorithm was used for spectral matching and decoy database construction^31–34^. Precursor ion mass tolerance was set to 50 ppm and fragment ion mass tolerance was set to 0.6 Da. The digesting enzyme was specified as trypsin, and two missed cleavages were allowed. The peptide length range was set between 6 and 144 amino acids. A dynamic modification for methionine oxidation was specified (+ 15.995 Da). Static modifications included isobaric tandem mass tags at peptide N-termini and on lysine residues (+ 229.163 Da). Carbamidomethylation of cysteine residues (+ 57.021 Da) was also specified as a static modification. A false discovery rate of 1% was used for filtering at the peptide and protein levels in Percolator. One mock-infected sample showed uncharacteristically low values and high rates of missing values in comparison to other samples. Thus, this sample was removed from subsequent analysis steps. Raw quantification values for the remaining samples were normalized against the average relative abundance values for each protein normalized against the median of all average values.

#### Proteomic statistical analyses

Statistical significance was determined using a π score threshold of 1^35^. The *p* value used to calculate the π score was determined by first performing an F test for equivalent variance between experimental conditions. If there was no significant difference in variance, the Student’s t-test was used to calculate the *p* value. If there was a significant difference in variance, the Student’s t-test with Welch’s correction was used to calculate the *p* value. All plots from the proteomic analysis were generated using Graphpad PRISM 7.

### Bioinformatic analysis

Gene ontology (GO) enrichment analysis of biological process and molecular function annotations were done using ClueGo app for Cytoscape (version 3.7.0). Also, upregulated and downregulated proteins were searched against the REACTOME Database using ClueGO app. An enrichment/depletion (two-sided hypergeometric test) method with Benjamini–Hochberg correction was performed. A minimum and maximal GO level of 3 and 8 were used, respectively. Kappa Score was set to 0.4.

### Statistical analysis

Data are presented as mean ± standard error of the mean (SEM). Statistical analyses used are one-way analysis of variance (ANOVA) or unpaired t-test as appropriate using Sigma Plot. Results were considered statistically significant at a value of *p* < 0.05.

## Data Availability

Raw spectral data can be accessed under the ProteomXchange identifier PXD014900.
